# A comparison of peak expiratory flow measured from forced vital capacity and peak flow meter manoeuvres in healthy volunteers

**DOI:** 10.4103/1817-1737.33697

**Published:** 2007

**Authors:** Dipti Agarwal, Prem Parkash Gupta

**Affiliations:** Department of Physiology, Postgraduate Institute of Medical Sciences, Rohtak, India; * *Department of Respiratory Medicine, Postgraduate Institute of Medical Sciences, Rohtak, India*

**Keywords:** Forced vital capacity maneuver, healthy volunteers, peak expiratory flow maneuver, peak expiratory flow rate, turbine spirometer

## Abstract

**BACKGROUND:**

Spirometry measures the mechanical function of lungs, chest wall and respiratory muscles by assessing the total volume of air exhaled from total lung capacity to residual volume. Spirometry and peak flow measurements have usually been carried out on separate equipments using different expiratory maneuvers.

**AIMS:**

The present study was carried out to determine whether there is a significant difference between peak expiratory flow (PEF) derived from a short sharp exhalation (PEF maneuver) and that derived from a full forced vital capacity (FVC) maneuver in healthy volunteers.

**SETTINGS:**

A medical college and tertiary level hospital.

**MATERIALS AND METHODS:**

The present study was carried out during the period from January 2006 to July 2006. The study included 80 healthy volunteers with no coexisting illnesses, who were in the 15-45 years age group and belonging to either sex. They were asked to perform two sets of PEF and FVC maneuvers using the same turbine spirometer; the order was randomly assigned.

**STATISTICAL ANALYSIS:**

The difference between PEF obtained from a peak flow maneuver (PEFPF) and that obtained from a forced vital capacity maneuver (PEFVC) in healthy volunteers was analyzed separately for males and females, as well as for both groups combined, and statistical significance of its correlations with study data parameters was analyzed.

**RESULTS:**

The difference between PEF obtained from a peak flow maneuver (PEFPF) and that obtained from a forced vital capacity maneuver (PEFVC) was statistically significant (*P* < 0.001) in males and in females separately and also for both groups combined. PEFPF (517.25 ± 83.22 liters/min) was significantly greater than PEFVC (511.09 ± 83.54 liters/min), as found on combined group mean analysis. However, the difference was small (6.16 + 7.09 liters/min).

**CONCLUSIONS:**

FVC maneuver can be used over spirometers to detect the PEF; and on follow-up subsequently, the same maneuver should be used to derive PEF. If we are using a peak flow maneuver subsequently, corrections are required to compensate for the difference due to the different maneuver.

The evaluation of human pulmonary functions dates back to the 17^th^ century. Borelli is considered to be the first physiologist who established the quantity of air received by a single inspiration.[1] In 1700, Humphrey measured the residual volume by hydrogen dilution technique using his ‘mercurial air-holding machine.’ Later, Hutchinson defined the functional subdivisions of lung volume.[1] For a century, progress in developing techniques for pulmonary function testing was sluggish. Since 1950s, however, pulmonary physiologists took advantage of the opportunities provided by the growing fields of electronics, transducers and computers; and since then, there has been tremendous progress in the arena of pulmonary function testing. Over the past 30 years, pulmonary function testing has been put to widespread clinical use and is presently considered an essential prerequisite to diagnose various obstructive and restrictive disorders. This has been made possible by several innovations: miniaturization and advancement in microprocessor devices, which have become portable, affordable and automated with fewer moving parts; testing equipments and techniques have been standardized through the efforts of professional societies; and, moreover, comprehensively accepted normative parameters have been established.

Peak expiratory flow (PEF) measurement is generally carried out using sophisticated spirometer using forced vital capacity maneuver in clinical setup, whereas at home the patients with chronic air flow obstruction are often advised to carry out their PEF measurement using simple (and affordable) peak flow meters as part of the patients' self-management strategies.[2] A discrepancy has been recognized between PEF measured by a portable peak flow meter and that measured by a spirometer in patients with CAO,[3] but whether the difference is related to recording equipment or dependent on the type of expiratory maneuver required is not clear.

The present study was carried out with the objective to determine whether there is a significant difference between PEF derived from a short sharp exhalation maneuver (peak expiratory maneuver) and that derived from a full forced vital capacity maneuver, using the same turbine spirometer in healthy volunteers.

## Materials and Methods

The present study was carried out in the Departments of Physiology and Respiratory Medicine at Postgraduate Institute of Medical Sciences, Rohtak, India, during the period from January 2006 to July 2006. A total of 80 healthy volunteers were included in the present study after offering an open invitation to the healthy volunteers through a local circular displayed on the notice boards at our institute.

### Study subjects

The subjects selected for the present study were in the age group of 15-45 years, belonging to either sex, and did not have any coexisting illness. The persons included were selected from amongst the medical students, medical/ paramedical staff of our institute and also amongst the healthy attendants of the patients attending the Respiratory Medicine Department of our institute. The healthy volunteers were recruited if they were found to have no respiratory or cardiovascular or neuromuscular illness after careful clinical assessment, spirometric investigations, chest radiographs and other relevant investigations required in individual cases. All healthy volunteers gave informed written consent for the study before their inclusion into the study.

### Study design

The pulmonary function tests were carried out on Vitalograph-Compact (UNISSI- India Pvt. Limited). As the subjects were not familiar with spirometry before the present study, they were given detailed instructions and also demonstration of the procedure before asking them to perform the spirometry. No medication - particularly the bronchodilators - was allowed within 24 h of performing the tests. They were allowed up to six attempts; and if they were unable to produce two blows <5% of maximum sum of FVC + FEV_1_, they were not included. They were asked to sit comfortably for at least 30 min before carrying out the second set of maneuver. The subjects were not permitted any medicine/ beverage/ cola drink/ meal/ physical activities between the two sets of maneuvers. The sets were performed in random order generated by the computer to exclude subjective bias. The PEF from PF maneuver was recorded by hand. Each FVC maneuver was saved electronically and printed out. For peak flow maneuver set, at least three maneuvers were performed until the two best PEF values were within 5%, and the PEF was selected from the best reading. For the vital capacity maneuver set, again, at least three maneuvers were carried out until the (FVC + FEV_1_) values were within 5% for the two best blows, and the maneuver with the greatest (FVC + FEV_1_) value was selected for PEF, in accordance with the ATS guidelines.[4]

### Statistical analysis

The statistical analyses for the study were carried out using the computer statistical software SPSS 10.0 version. Initially, the data was analyzed including males and females in two different groups; later, the analyses were done considering them as a single group. Analyses of physical characteristics were carried out using descriptive statistics; an analysis of study parameters was carried out using the paired sample ‘t’ test. In addition, the correlations between the difference in PEF measured by different maneuvers and the subjects' characteristics - age, height, FEV_1_, FVC and FEV_1_/FVC ratio - were ascertained using Pearson's correlation test.

## Results

A total of 80 healthy volunteers completed the study according to the study protocol; out of which, 46 [57.5%] were male and 34 [42.5%] were female - with a male/ female ratio of 1.35: 1. This was probably due to more male subjects being available for voluntary inclusion into the study, which in no way reflects any gender-specific significance or any bias.

**Figure 1:**
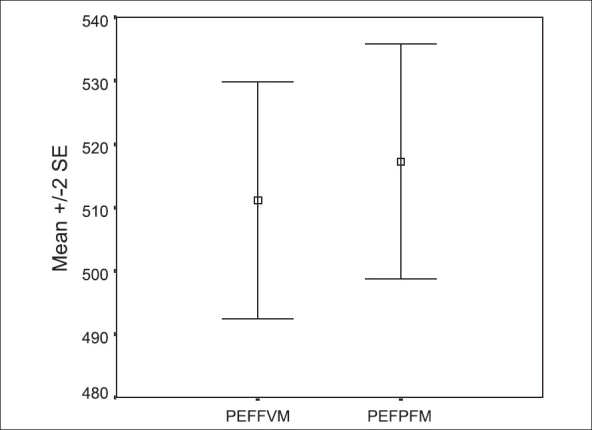
The mean and SEM of PEF (in liters/min) by forced vital capacity maneuver vs. PEF (in liters/min) by peak flow maneuver Graphical representation of the mean as well as the standard error of mean (mean ± 2SE) for [i] PEFVM - flow volume maneuver; and [ii] PEFPFM – the peak expiratory flow maneuver

The physical characteristics regarding age, height and other parameters of male subjects, female subjects and both of them combined are as shown in Table 1. Within the study group, paired sample ‘t’ test analyses were carried out to investigate the statistical significance of the difference between two sets of maneuvers [peak flow maneuver (PEFPF) and forced vital maneuver (PEFVC)]. The difference between PEF by peak flow maneuver and that by forced vital maneuver was 6.40 ± 6.33 liters/min in the male group, 5.84 ± 8.08 liters/min in female group and 6.16 ± 7.09 liters/min in both groups combined together. Though the differences were small, they were statistically highly significant (*P* < 0.001). Figure 1 demonstrates the error bar to illustrate the mean and standard error of PEF by forced vital capacity maneuver *vs.* PEF by peak flow maneuver.

**Table 1:** Study data

Groups 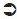	Males	Females	Both groups combined
No. of subjects	46	34	80
Age, years [Mean ± SD]	29.30 ± 7.74	29.65 ± 7.70	29.45 ± 7.67
Height, cm [Mean ± SD]	168.83 ± 3.93	156.82 ± 5.69	163.73 ± 7.61
FEV_1_, liters [Mean ± SD]	3.644 ± 0.242	2.516 ±0.194	3.164 ± 0.6035
FVC, liters [Mean ± SD]	4.534 ± 0.259	3.135 ± 0.188	3.939 ± 07333
PEF by Peak expiratory flow Maneuver, liters/min [Mean ± SD]	586.70 ± 20.37	423.29 ± 13.39	517.25 ± 83.22
PEF by forced vital capacity Maneuver, liters/min [Mean ± SD]	580.29 ± 23.15	417.45 ± 16.29	511.09 ± 83.54
PEF by peak flow maneuver - PEF by forced vital maneuver, liters/min [Mean ± SD]	6.40 ± 6.33	5.84 ± 8.08	6.16 ± 7.09
Statistical significance of difference between	*P*<0.001	*P*<0.001	*P*<0.001
PEF by two different maneuvers	[significant]	[significant]	[significant]

The analyses were also done to see the correlations between the difference in PEF measured by two different maneuvers and the patients' characteristics, viz., age, height, FEV_1_, FVC and FEV_1_/FVC ratio [Table 2]. For correlation study, the data from both groups was combined together. Out of various variables, only correlation between the height of the subject and the difference in PEF by peak expiratory maneuver and that by forced vital maneuver was statistically significant (*P* = 0.004); the correlation was a negative one.

**Table 2:** Correlation study

	Age	Height	FEV1	FVC	FEV1/FVC ratio
Pearson's correlation	0.113	-0.323	-0.131	-0.121	-0.100
Sig. (2-tailed)	0.317	0.004	0.247	0.286	0.377
Statistical significance	Not significant	Significant	Not significant	Not significant	Not significant

Correlation is shown between the difference if PEF measured by different maneuvers [PEF by peak flow maneuver - PEF by forced vital maneuver] and various study parameters as shown in the first row. For correlation study, the data from both groups was combined together.

## Discussion

Spirometry is the most widely used screening test for lung function or pulmonary function studies. It is usually the first test to be performed and interpreted. Spirometry can be carried out in the ambulatory setting, physician's office, emergency department or inpatient setting. Electronic spirometry provides much more information about airway function while still providing PEF measurements. Invariably, peak expiratory flow measurement is carried out using a sophisticated spirometer using forced vital capacity maneuver in clinical setup. The patients' self-management strategies[2] require the active involvement of patients in monitoring their lung functions at home, in order to be more confident in the management of their disease as well as to initiate early additional medications in case of exacerbations. At home, the patients with chronic air flow obstruction who carry out their PEF measurement usually do so using peak flow meters; these measurements are not only simple to perform and easy to evaluate but are also more affordable. Discrepancies have been demonstrated[3] between PEF measured by portable PEF meters and that measured using spirometry. Uwyedd *et al.* studied children with asthma to assess the contribution of PEF monitoring at home to asthma management.[3] They found poor agreement between PEF from meter and PEF from spirometer and concluded that ‘PEF recorded by a mini-Wright meter does not necessarily reflect that recorded by spirometer.’ Hankinson *et al.*[5] postulated that PEF meters overestimate PEF at lower flow rates when compared to spirometry. A variable error in PEF measurements obtained using mini-Wright meters has also been described.[6]

Some workers have advocated spirometry in place of PEF meters for monitoring adults and children with asthma at home.[7,8] However, little work has been done to determine whether the difference in PEF from PEF meter and PEF from spirometer is related to the recording equipment itself or dependent on the type of forced expiratory maneuver required. After a careful search for published studies in medical journals and over internet using PubMed website (www.ncbi.nih.gov/entrez) and Highwire website (www.highwire.org), we could not find a study in healthy adults addressing this issue. A study by Wensley and co-workers[9] included 80 children (38 with current asthma) aged 7 -16 years. They found PEF obtained from a peak flow maneuver was significantly greater than that obtained from a forced vital capacity maneuver in both healthy and asthmatic children. The overall mean difference was about 5%; and for the children with asthma, it was 3%.

In the present study, we observe a small but statistically highly significant difference between peak expiratory flow by peak flow maneuver and that by forced vital maneuver while using the same turbine spirometer for both maneuvers. The difference between them was small and clinically insignificant as per guidelines.[10] The implication from our study is that FVC maneuver can be used over spirometers to detect the PEF; and on follow-up subsequently, the same maneuver (and probably the equipment also) should be used to derive PEF. However, if we are using a different maneuver (i.e., peak flow maneuver) to record PEF subsequently, corrections are required to compensate for the difference due to the different maneuver.
